# Exosomal microRNAs: implications in the pathogenesis and clinical applications of subarachnoid hemorrhage

**DOI:** 10.3389/fnmol.2023.1300864

**Published:** 2023-12-07

**Authors:** Lishang Liao, Haoran Wang, Deli Wei, Mingliang Yi, Yingjiang Gu, Mingwei Zhang, Li Wang

**Affiliations:** ^1^Department of Neurosurgery, The Affiliated Traditional Chinese Medicine Hospital of Southwest Medical University, Luzhou, China; ^2^Department of Neurosurgery, The People’s Hospital of Fushun County, Zigong, China; ^3^Department of Research Center of Integrated Traditional Chinese and Western Medicine, The Affiliated Traditional Chinese Medicine Hospital of Southwest Medical University, Luzhou, China

**Keywords:** subarachnoid hemorrhage, early brain injury, exosomal micro-RNAs, apoptosis, inflammatory response, immune activation

## Abstract

Subarachnoid hemorrhage (SAH) is a severe acute neurological disorder with a high fatality rate. Early brain injury (EBI) and cerebral vasospasm are two critical complications of SAH that significantly contribute to poor prognosis. Currently, surgical intervention and interventional therapy are the main treatment options for SAH, but their effectiveness is limited. Exosomes, which are a type of extracellular vesicles, play a crucial role in intercellular communication and have been extensively studied in the past decade due to their potential influence on disease progression, diagnosis, and treatment. As one of the most important components of exosomes, miRNA plays both direct and indirect roles in affecting disease progression. Previous research has found that exosomal miRNA is involved in the development of various diseases, such as tumors, chronic hepatitis, atherosclerosis, diabetes, and SAH. This review focuses on exploring the impact of exosomal miRNA on SAH, including its influence on neuronal apoptosis, inflammatory response, and immune activation following SAH. Furthermore, this review highlights the potential clinical applications of exosomal miRNA in the treatment of SAH. Although current research on this topic is limited and the clinical application of exosomal miRNA has inherent limitations, we aim to provide a concise summary of existing research progress and offer new insights for future research directions and trends in this field.

## 1 Introduction

Subarachnoid hemorrhage (SAH) is a severe stroke event that has an alarmingly high mortality and disability rate (Lawton and Vates, 2017), accounting for approximately 3–5% of the total incidence of stroke (Vivancos et al., 2014; Lai et al., 2017). It is caused by blood flow into the subarachnoid space after rupture of intracranial blood vessels. It can be classified as traumatic or non-traumatic and result from aneurysm, hypertension, smoking, alcoholism, drug abuse, among other factors (Bakker et al., 2020; Karhunen et al., 2021; Wang et al., 2021a). SAH results in a sudden increase in intracranial pressure, leading to systemic ischemia and hypoxia, ultimately causing brain tissue damage and various neurological dysfunctions (Fang et al., 2019). Extant research indicates that EBI and cerebral vasospasm are the two primary complications of SAH (Angermann et al., 2022; Zhao et al., 2022). The current treatment options mainly involve surgery and interventional therapy, which often only address symptomatic and hemostatic treatment. Additionally, there remain several significant limitations in identifying, preventing, and correcting complications (Chung et al., 2022; Endo et al., 2022). Therefore, it is crucial to apply advanced technology and tools for early identification and intervention during the early stage of SAH.

Exosomes are extracellular vesicles that range in size from 60 to 120 nm (Bobrie et al., 2012). They are primarily derived from the invagination and maturation of early endosomes into late endosomes or multivesicular bodies (MVBs). These MVBs, bearing specific markers like CD63, LAMP1, LAMP2, and MHC class II in antigen-presenting cells, can fuse with the plasma membrane (PM) to release exosomes (Colombo et al., 2014). Almost every type of cell has the ability to secrete exosomes, which can be found in a broad range of body fluids, such as blood, urine, cerebrospinal fluid, saliva, and milk (Ludwig and Giebel, 2012). As one of the carriers of intercellular communication, exosomes are rich in various contents, with microRNA (miRNA) being one of the most critical substances. Exosomal miRNAs are a type of non-coding RNAs that can regulate an extensive array of biological processes. By inhibiting or promoting the transcription and translation of target genes, exosomal miRNAs can affect cell biological functions and play a crucial role in intercellular signaling, cell growth and differentiation, being considered a unique type of regulatory molecules (Garcia-Martin et al., 2022). In the past decade, molecular research has shown that exosomal miRNAs impact the development and progression of an array of diseases, such as metabolic diseases (Castaño et al., 2018; Xu et al., 2022b), tumors (Cariello et al., 2022), hepatitis (Qiu et al., 2022), renal system diseases (Zhao et al., 2021a), cardiovascular diseases (Du et al., 2021), among others, significantly contributing to potential clinical applications.

It is essential to note that exosomal miRNAs have been widely observed to exert a significant impact on the occurrence and development of SAH, significantly influencing neuronal apoptosis, cerebral microcirculation disorders, and neuroinflammation (Zhao et al., 2019; Lai et al., 2020; Xiong et al., 2020). They are expected to become a novel generation of molecular diagnostic markers (Sheng et al., 2023). This article summarizes the correlation between exosomal miRNA and SAH, as well as their potential benefits and application value for clinical diagnosis and treatment compared to conventional methods. Additionally, it identifies the limitations of existing research and highlights directions for future research.

## 2 Subarachnoid hemorrhage

Subarachnoid hemorrhage, classified as either traumatic or spontaneous, is the third most common subtype of stroke. A meta-analysis conducted in 2018, which included 75 studies from 32 countries, found that spontaneous SAH occurs in approximately 8 per 100,000 individuals per year (Hughes et al., 2018). The incidence of SAH has been declining over the past few decades, which may be attributed to lifestyle changes such as smoking cessation and better management of hypertension. However, the age of onset and the poor prognosis of SAH significantly impact the quality of life of patients for many years, making it a crucial public health concern (Claassen and Park, 2022).

The process of SAH involves various cellular and molecular mechanisms, which, when combined, significantly contribute to the development of complications and poor prognosis. Firstly, during bleeding, the accumulation of red blood cells and platelets in the subarachnoid space results in the formation of a blood clot. This triggers the activation of the coagulation system and inflammatory response. This, in turn, leads to the release of various inflammatory factors and cytokines, including tumor necrosis factor-alpha (TNF-α), interleukin-1beta (IL-1β), and interleukin-6 (IL-6), among others. Consequently, the brain tissue’s sensitivity to injury increases, exacerbating neuronal death and the inflammatory response. Ultimately, this results in a poor prognosis (Fragata et al., 2020; Devlin et al., 2022). Secondly, SAH causes an increase in pressure in the subarachnoid space, leading to mechanical damage to brain tissue. This mechanical damage disrupts the integrity of cell membranes, resulting in cell death and dysfunction. Additionally, bleeding and hematoma compression hinder proper blood circulation, causing brain tissue hypoxia and further impairing cell function (Marcolini et al., 2019). Thirdly, SAH initiates a series of physiological and pathological processes, including the release of excitatory amino acids, apoptosis, oxidative stress, and disruption of the blood-brain barrier (BBB). These processes activate multiple signaling pathways and cytokines, ultimately resulting in an inflammatory response and cell death (Mo et al., 2019).

Overall, the abnormal responses observed in SAH are closely associated with molecular and cellular mechanisms, playing a significant role in the development of complications and poor prognosis. A thorough investigation of these mechanisms will enhance our understanding of the disease onset and progression, guiding the search for more effective treatments.

## 3 Exosome and exosomal miRNA

### 3.1 Biogenesis of exosomes and exosomal miRNAs

The biogenesis of exosomes and the exosomal miRNAs involves intricate interactions among various organelles and molecules. This complex process is influenced by both intra- and extracellular factors, such as intracellular signaling, secretion mechanisms, and intercellular information exchange (Hannafon and Ding, 2013). Gaining a comprehensive understanding of this process is essential for elucidating the functional roles of exosomes and exosomal miRNAs and harnessing their potential in diagnostic and therapeutic applications.

The initial discovery of miRNAs dates to 1993 in C. elegans when their importance in regulating post-embryonic developmental events was recognized (Lee et al., 1993). MiRNAs are small RNA molecules composed of approximately 22 nucleotides that primarily regulate gene expression by binding to target mRNAs (Lai et al., 2003; Lim et al., 2003; Bartel, 2004). They exert their regulatory effects post-transcriptionally and participate in various biological processes, including cell cycle regulation, cell differentiation and development, immune response, and tumorigenesis (Calin et al., 2004; Tsuchiya et al., 2006; Liu, 2008). Extracellular miRNAs are released from cells and enter the bloodstream or other bodily fluids, where they can be encapsulated within extracellular vesicles such as exosomes, microvesicles, or high-density lipoproteins (HDL) (Mori et al., 2019; Groot and Lee, 2020). They play a crucial role in intercellular communication and information transmission by binding to receptors on target cells. This allows them to be internalized or adsorbed onto the surface of recipient cells, thereby mediating intercellular regulation and communication (Makarova et al., 2021). Through the modulation of gene expression, extracellular miRNAs are believed to influence the function and status of recipient cells. Research has discovered the crucial role of extracellular miRNAs in both physiological and pathological processes, including immune regulation, tissue regeneration, and embryonic development. Moreover, abnormal expression of extracellular miRNAs has been observed in various diseases, indicating their potential as biomarkers and therapeutic targets for disease diagnosis, prediction, and treatment (Backes et al., 2016; Yoshioka et al., 2018; Conti et al., 2020).

The biogenesis of exosomal miRNAs begins in the nucleus where RNA polymerase II transcribes miRNA genes into primary miRNAs (pri-miRNAs). These pri-miRNAs subsequently form a characteristic hairpin structure called precursor miRNA (pre-miRNA) (Ramachandran and Palanisamy, 2012). Afterward, an RNase III enzyme called Drosha and its cofactor DGCR8 recognize and cleave this pre-miRNA in a process known as pre-miRNA processing. This step results in generating a matured pre-micro-RNA that is then transported from the nucleus to cytoplasm via exportin-5/Ran-GTP complex. This transportation ensures that it reaches its destination for further processing (Lee et al., 2012). Upon entering the cytoplasm, pre-miRNA is identified and cleaved by another RNase III enzyme called Dicer in conjunction with its partner protein TRBP. This cleavage event results in forming a double-stranded miRNA: miRNA* duplex that is typically around 22 nucleotides long (Tran, 2016). One of these strands functions as mature miRNA and integrates into RNA-induced silencing complex (RISC), while referred to as passenger strand (miRNA*) usually undergoes degradation. Once integrated into RISC complex, mature miRNA guides recognition and binding of target mRNAs which subsequently leads to their degradation or translational repression (Zhang et al., 2015). Furthermore, a portion of mature miRNAs may also be selectively loaded into MVBs, which function as precursors to exosomes. These MVBs containing loaded mature miRNAs then bud inward from the endosomal membrane, resulting in the formation of intraluminal vesicles (ILVs) (Tamai et al., 2010; Wollert and Hurley, 2010). Ultimately, these ILVs, which are composed of exosomes, are released into the extracellular environment upon fusion of MVBs with the plasma membrane (Sun et al., 2018).

### 3.2 Characteristics of exosomes and exosomal miRNAs

Exosomes, one of the three main kinds of extracellular vesicles alongside microvesicles and apoptotic bodies (Théry et al., 2009), can be divided into two subgroups based on their size under electron microscopy: large exosome vesicles (Exo-L) with a size between 90 and 120 nm, and small exosome vesicles (Exo-S) ranging in size from 60 to 80 (Zhang et al., 2018). In virtually all mammalian cells, exosomes are ubiquitous and can be absorbed and secreted (Kowal et al., 2014; Munson and Shukla, 2015). Fat cells (Thomou et al., 2017), immune cells (Raposo et al., 1996; Zitvogel et al., 1998), brain cells (Zhang et al., 2017), and tumor cells (Luo et al., 2022; Wang et al., 2022a; Xu et al., 2022a) are among the cells capable of generating exosomes. Exosomes naturally occur in various body fluids, including serum, saliva, cerebrospinal fluid, urine and seminal fluid (Pisitkun et al., 2004; Akers et al., 2013; Vojtech et al., 2014; Hornick et al., 2015). Exosomes have been shown to contain nucleic acids, proteins, lipids, and various metabolic products (Valadi et al., 2007; Zhao et al., 2016). Furthermore, apart from their cargo of specific proteins, exosomes also contain a range of non-specific proteins that serve various functions. These proteins include membrane fusion and transfer proteins, major histocompatibility complex (MHC) proteins, heat shock proteins, and cytoskeleton proteins (Wang et al., 2015; Bi et al., 2022). These substances play a crucial role in intercellular communication and are indispensable for normal physiological functions of tissues and cells (Wu et al., 2022).

### 3.3 Isolation, extraction, and preparation of exosomes

Exosomes serve as carriers of exosomal miRNAs, and their potential clinical application is closely linked to the utilization of exosomal miRNA. To widely employ exosomal miRNAs in clinical studies, attention must be given to the isolation, extraction, and preparation of exosomes.

Over the years since the discovery of exosomes from reticulocytes in 1983, multiple methods for separating exosomes have emerged and gradually matured to cater to the specific requirements of this field (Johnstone et al., 1987). Differential ultracentrifugation was the initial method employed for exosome separation and remains the gold standard due to its simplicity, convenience, cost-effectiveness, and efficacy (Hiemstra et al., 2014; Muller et al., 2014; Livshits et al., 2015). Similar to density gradient centrifugation, this technique relies on centrifugal force to separate substances based on their density and size (Li et al., 2017). However, both methods suffer from low exosome recovery rates, rendering them inadequate for large-scale clinical applications and limiting their usage to certain laboratories. Consequently, the emergence of molecular immunotechnology has brought about groundbreaking changes in exosome separation methods. Enzyme-linked immunosorbent assay (ELISA), commonly employed in clinical practice for virus detection through immunoaffinity capture, has not been extensively applied in exosome separation due to additional requirements such as sample ultrafiltration or ultracentrifugation (Mathivanan et al., 2010). Anyway, this led to the application of the magnetic immunoprecipitation method, which significantly improved separation efficiency, enabled handling of larger sample volumes, and retained exosome activity (Hong et al., 2014). Furthermore, the application of nanotechnology has led to another breakthrough in exosome separation technology. Acoustic nanofilters utilize ultrasonic radiation force to separate and extract exosomes based on their varying sizes and densities. Although still in the developmental stage, this method shows promise due to its simplicity, speed, adjustability, and ability to work with smaller sample volumes, positioning it as a potentially valuable tool in clinical settings (Lee et al., 2015).

The increasing demand for analyzing exosomes in both basic research and clinical studies has led to the development of various *in vitro* validation methods. *In vitro* validation typically involves quantitative and qualitative analyses, each with their own distinct advantages and applications.

The quantitative analysis of exosomes centers on determining the quantity of exosomes present. Notable techniques for this type of analysis include nanoparticle tracking analysis and flow cytometry. Nanoparticle tracking analysis (NTA) employs high-throughput visualization to determine particle size and concentration by analyzing the Brownian motion of particles under laser illumination (Dragovic et al., 2011; Kanwar et al., 2014). On the other hand, flow cytometry involves labeling exosomes, and using this labeled data to collect and analyze information regarding exosome count, fluorescence intensity, surface markers, and more (Szatanek et al., 2017). This method enables the assessment of exosome production and release rates, making it suitable for studying exosome changes under different conditions and variations between cell types or disease states.

The qualitative analysis of exosomes aims to identify their composition and function. Techniques such as mass spectrometry, electron microscopy, immunostaining, and the examination of morphology and structure provide valuable insights into the protein, nucleic acid, lipid, and other components of exosomes (Doyle and Wang, 2019). Additionally, functional analysis involves conducting experiments to verify the interactions between exosomes and recipient cells, such as binding experiments that assess the attachment of exosome inclusions to receptors on cell surfaces (Cianciaruso et al., 2019). Qualitative analysis provides detailed information about exosome composition and function, facilitating a deeper understanding of their role in intercellular communication and disease development.

These methods can be employed individually or in combination to determine the presence, quantity, composition, and function of exosomes. The development of rapid, high-throughput, and reproducible exosome detection methods is crucial for utilizing circulating exosomes as biomarkers.

### 3.4 Relationship between biological function of exosomal miRNAs and human diseases

One of the fascinating features of exosomal miRNAs is their ability to transfer genetic information between cells. When recipient cells take up exosomes, miRNAs are released and can modulate gene expression by binding to the 3′ untranslated region (UTR) of target mRNAs (Askenase, 2021; Garcia-Martin et al., 2022). This regulatory mechanism mediated by miRNAs plays a critical role in influencing diverse cellular processes, such as proliferation, differentiation, apoptosis, and immune response (Fiorani et al., 2023). It is noteworthy that exosomal miRNAs not only exert paracrine effects within the local microenvironment but also exhibit endocrine effects, facilitating long-distance communication between cells and impacting the behavior of distant cells throughout the body (Rahimian et al., 2023).

Extensive research has been conducted in recent years to explore the impact of exosomal miRNAs on various diseases. In the context of tumors, exosomal miRNAs have been found to influence tumor cell proliferation and mobility, contribute to the creation of a tumor-suppressing immune microenvironment, and play a role in establishing blood supply within tumor tissues (Zhao et al., 2020, 2021b; Wang et al., 2021b). In metabolic disorders, exosomal miRNAs are involved in the regulation of glucose and lipid metabolism, potentially leading to conditions such as obesity and diabetes (Castaño et al., 2018; Abbas et al., 2023). Additionally, exosomal miRNAs play a role in regulating neuronal apoptosis, neuronal inflammation, and cerebrovascular development in diseases that affect the nervous system (Sun et al., 2019; Lai et al., 2020; Li et al., 2022; Wang et al., 2022b). Moreover, due to their unique composition and stability within exosomes, miRNAs are ideal candidates for diagnostic purposes and monitoring disease progression (Salehi and Sharifi, 2018; Aghabozorgi et al., 2019).

**FIGURE 1:**
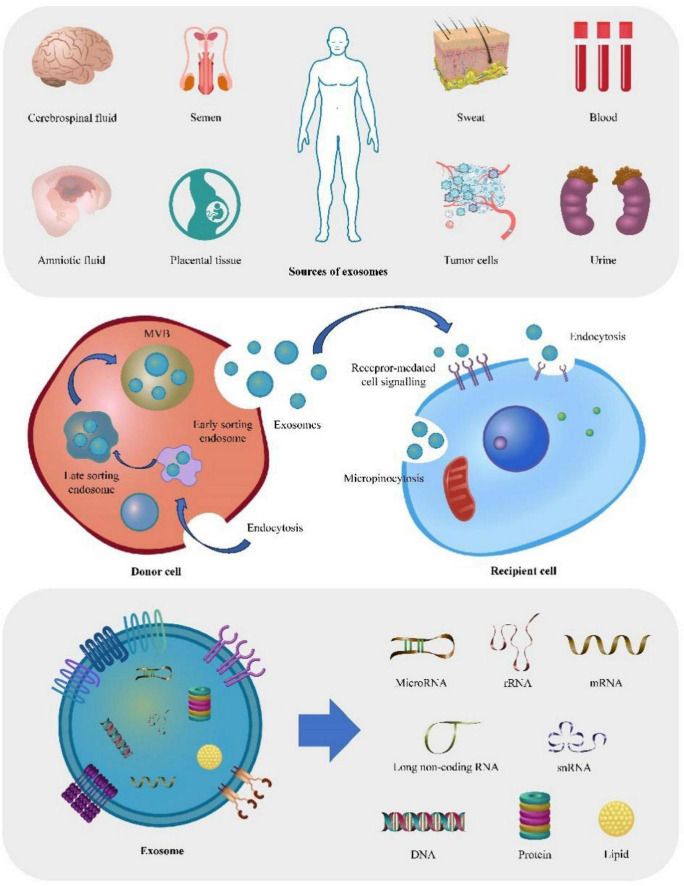
Depict the origin, production mechanism, action process, and contents of exosomes. The figure offers a comprehensive overview of exosomes, starting from their potential sources. Firstly, exosomes can be secreted by virtually all tissues or organs, and can therefore be detected in various bodily fluids, including blood, saliva, and sweat. Furthermore, the figure illustrates the process of exosome production, wherein early endosomes undergo invagination and maturation, ultimately transforming into late endosomes or multi-vesicular bodies (MVBs). Lastly, the figure highlights the typical contents found within exosomes, including DNA, RNA, lipids, and proteins.

In summary, exosomes can have both beneficial and detrimental effects on human health. Among the cargo carried by exosomes, miRNAs have emerged as key players in regulating gene expression in recipient cells. The innovative use of exosomes and their cargo of miRNAs holds the potential for revolutionary advancements in the diagnosis and treatment of various diseases (Figure 1).

## 4 The role and mechanism of exosomal miRNA in SAH

Exosomal miRNAs play a critical role in the pathogenesis of SAH. Notable examples that highlight their significance in this context are summarized here, providing insights into their role and clinical importance in the progression of SAH (Table 1).

**TABLE 1:** Present a summary of the effects of different exosomal miRNA on SAH.

Exosomal miRNA	Influencing mechanism	Resource	Result	Clinical significance
miR-124 (Chen et al., 2020)	It has been observed that the targeting protein CCAAT enhancer binding protein α can effectively inhibit the activation of microglial cells.	Embryonic neuron	Anti-inflammatory and neuroprotective effects.	Promoting the delivery of miR-124 to the microglia presents itself as a potential novel approach to treating SAH-induced EBI
miR-193b-3p (Lai et al., 2020)	Suppress the expression and the activity of HDAC3 and increase NF-κB p65 acetylation after SAH	Blood	Reduce neuroinflammation and neuronal degeneration	Potential methods to reduce neuroinflammation in EBI after SAH
miR-129-5p (Xiong et al., 2020)	The activity of HMGB1-TLR4 pathway was inhibited	BMSCs	Anti-inflammatory and anti-apoptotic effects	Alleviate early brain damage after SAH
miR-206 (Zhao et al., 2019)	Neuronal apoptosis was inhibited by BDNF/TrkB/CREB signaling pathway	Huc-MSCs	To prevent EBI by inhibiting neuronal apoptosis	It can be used as a new therapeutic target for SAH-induced EBI
miR-26b-5p (Liu et al., 2021)	The p38 MAPK/STAT3 signaling pathway mediated by MAT2A	Hub-MSCs	Brain cell apoptosis, neuronal necrosis, albumin exudation and cerebral edema	Provide a new idea for alleviating EBI
miR-140-5p (Wang et al., 2022b)	Regulate IGFBP5-Mediated PI3K/AKT Signaling Pathway	Tumor cells	Inhibition of neuronal apoptosis	Reduce neuronal damage and neurological dysfunction
miR-3064-5p (Wang et al., 2023)	MiR-3064-5p can target the inhibition of Sirtuin 6 (SIRT6), thereby reducing the level of cellular inflammatory factors and inhibiting cell apoptosis	Dendritic cells	Protect the BBB	It may be a targeted drug and method to protect the BBB

### 4.1 Exosomal miRNA mitigates apoptosis and restores damaged neurons

Neuronal apoptosis is closely associated with oxidative stress following SAH, with the cerebral cortex and hippocampus being the primary affected areas (Ayer and Zhang, 2008). As neurons are post-mitotic cells and most neurons cannot be replaced in the adult central nervous system after injury, the rate and number of neuronal apoptosis can affect the prognosis and recovery of brain function in SAH patients. Therefore, early and targeted interventions and management are crucial for reducing adverse outcomes.

Exosomal miRNAs derived from different cell sources exhibit varying effects on SAH. Microglia, as essential immune cells within the central nervous system (CNS), make up approximately 10% of the total cell population and serve as a primary defense against CNS damage. After SAH, microglia activation-mediated neuroinflammation becomes the main contributor to EBI. A neuron-derived miRNA-124, assumes a crucial role in regulating microglial activation by targeting the protein CCAAT enhancer binding protein α (C/EBPα). This molecular interaction plays a crucial role in reducing neuronal death following SAH (Chen et al., 2020). Additionally, exosomal miR-140-5p derived from adipose tissue has the ability to inhibit TDP-3-induced neuronal apoptosis by targeting and inhibiting IGFBP43, while simultaneously activating the PI3K/Akt signaling pathway. These findings highlight the potential of exosomal miR-140-5p in ameliorating neural dysfunction and hold promise for therapeutic intervention (Wang et al., 2022b).

The results of these studies indicate that exosomal miRNAs have significant potential in reducing neuronal apoptosis, promoting neuronal repair, and modulating adverse outcomes related to SAH. The distinct characteristics of exosomal miRNAs derived from various cell sources enable the specific targeting of therapeutic pathways involved in the development of SAH. Further investigations are expected to reveal novel exosomal miRNAs with unique functionalities, thereby expanding our knowledge of their therapeutic application in SAH treatment.

### 4.2 Exosomal miRNA restraint neuroinflammation and immune activation

Neuroinflammation involves inflammation of nerve tissue, which is known to cause brain injury, stroke, brain bleeding, and other traumatic events that can significantly harm brain function (Lyman et al., 2014; Yong et al., 2019). Several pathological processes after SAH result in the activation of an inflammatory cascade, leading to damage to the BBB and degeneration of neurons (Lan et al., 2019). Moreover, SAH provokes the activation of numerous immune cells, which subsequently release inflammatory mediators, triggering an inflammatory response in nerve tissue and, consequently, exacerbates neurological dysfunction (Kumar et al., 2019; Li et al., 2021a).

MiR-124, a highly abundant miRNA within the brain (Mishima et al., 2007), was initially discovered in Lagos-Quintana et al. (2002). To date, three human miR-124 subtypes have been identified (Qin and Liu, 2019). It is predominantly synthesized by glial cells, followed by non-glial cells and immune cells in certain peripheral tissues. By regulating transcription factors and signaling pathway-related genes, miR-124 plays a vital role in neuronal differentiation, axon growth, and synapse formation (Baroukh and Van Obberghen, 2009). Abnormal expression of miR-124 has been observed in various neurological disorders and tumors, including Alzheimer’s disease (Ouyang et al., 2022), Parkinson’s disease (Esteves et al., 2022), SAH (Chen et al., 2020), amyotrophic lateral sclerosis (ALS) (Vaz et al., 2021), colorectal cancer (Shahmohamadnejad et al., 2022), and glioma (Hong et al., 2021). Microglia, a crucial type of immune cell, significantly contribute to neuroinflammation, which is considered a primary factor in EBI following SAH (Prinz et al., 2019). Elevating miR-124-3p levels in microglia-derived exosomes after traumatic brain injury could inhibit neuroinflammation and promote neurite growth by transferring miR-124-3p to neurons (Huang et al., 2018). In a rat stroke experiment, extracellular vesicles containing miR-124, released by microglia, targeted the STAT3 pathway to mitigate glial scar formation, demonstrating potential for addressing neuronal regeneration disorders post-stroke (Li et al., 2021b). Astrocytes, the most prevalent type of glial cell in the central nervous system, play a critical supportive and regulatory role, essential for maintaining neuronal function and environmental homeostasis (Freeman, 2010). Interestingly, neuron-derived miR-124 induces direct conversion of active astrocytes into immature neurons *in vivo* (Papadimitriou et al., 2023). Furthermore, miR-124-3p delivered via neuron-derived exosomes can safeguard the injured spinal cord by inhibiting the activation of neurotoxic microglia and astrocytes (Jiang et al., 2020). However, the study of exosomal miRNA derived from astrocytes in the context of SAH is still limited, necessitating further research progress.

An analogous outcome can be achieved by activating signaling pathways implicated in the inflammatory cascade. In response to passive injury, the NF-κB signaling pathway becomes active within cells, triggering microglia activation and subsequent secretion of inflammatory cytokines, thereby amplifying the inflammatory response cascade. However, the presence of exosome miR-193b-3p can hinder the HDAC3/NF-κB signaling pathway, resulting in a reduction of neuroinflammation and conferring neuroprotective effects (Lai et al., 2020).

### 4.3 Exosomal miRNA facilitates cerebrovascular formation and assists in restoring cerebral microcirculation

Cerebral microcirculation is primarily based on the BBB’s structural and functional basis, comprising vascular endothelial cells, endothelium-cell junctions, the basement membrane, and astrocytes (Kondo et al., 2022; Yang et al., 2022; Hu et al., 2023). Recently, considerable attention has been given to the role of cerebral microcirculation disturbances in EBI following SAH. The potential mechanisms for this pathology include inflammation, oxidative stress injury, platelet activation, long-term vasoconstriction, and endothelial cell apoptosis (Yu et al., 2015).

In an *in vitro* study aimed at mimicking the extracellular environment seen after SAH, exposure of brain microvascular endothelial cells (BMECs) to a cell culture medium composed of Blood-Cerebrospinal Fluid (BCSF) pointed to a crucial role of exosomal miR-630 in SAH. Notably, the researchers observed a significant decrease in the quantity of exosomal miR-630 in BMECs treated with BCSF, which was consistent with the change of exosome miRNA-630 in cerebrospinal fluid of patients with SAH. The research team further discovered that BMECs co-cultured with exosomes transfected with miR-630 mimics showed significantly increased expression levels of ICAM-1, VCAM-1, and the tight junction protein ZO-1 compared to the control group. These findings suggest that exosome miR-630 might regulate cell adhesion and the function of tight junctions in BMECs, which could contribute to improving brain microcirculation. However, the specific regulatory mechanism and downstream targets of exosomal miR-630 require further investigation (Sun et al., 2019). Additionally, an investigation was conducted to compare the profiles of cerebrospinal fluid miRNAs in patients with and without cerebral vasospasm, a severe complication characterized by the constriction of brain blood vessels following a hemorrhage. The analysis revealed significant differences in the levels of miR-27a-3p, miR-516a-5p, miR-566, and miR-1197 between the two groups (Stylli et al., 2017). These findings highlight the vital role of exosomal miRNAs in the development of cerebrovascular structures and the regulation of cerebral microcirculation.

Despite these notable advancements, there is an urgent need for additional groundbreaking research in these domains. A comprehensive elucidation of the intricate molecular mechanisms governing exosomal miRNA regulation and its downstream effects on cerebrovascular formation and cerebral microcirculation holds immense potential for advancing our understanding of SAH complications. Furthermore, such research endeavors may pave the way for developing innovative therapeutic strategies to effectively manage and treat SAH-related complications. Addressing these existing research gaps is crucial in our pursuit of enhanced preventive measures, accurate diagnoses, and more efficient treatment options to benefit patients suffering from SAH complications.

## 5 Clinical significance of exosomal miRNA in SAH

### 5.1 Exosomal miRNA hold diagnostic and prognostic value

The diagnosis and management of SAH face limitations due to the lack of pathophysiological features and molecular markers accurately and timely reflecting the disease (Chen et al., 2014). Exosomes exist widely in peripheral blood and cerebrospinal fluid, and their contents can be used to detect their presence. The emergence of second-generation sequencing technology has enabled the detection of differences in exosomal miRNA, which are becoming increasingly important biomarkers for diagnosing and prognosticating SAH (Thakur et al., 2014).

Intracranial aneurysm rupture is a leading cause of SAH. In a study performed to assess the predictive significance of extrinsic miRNAs in SAH, the level of these miRNAs was compared in the CSF of 152 patients with unruptured intracranial aneurysms, three patients with ruptured intracranial aneurysms, and 66 patients with hydrocephalus. The study revealed that exosomal miR-603 had significantly higher concentrations in the cerebrospinal fluid of patients with SAH, which affected vascular smooth muscle cell dysfunction (Li et al., 2022). Moreover, Bing Sheng et al. discovered that exosomal miR-369-3p, miR-410-3p, miR-193b-3p, and miR-486-3p might influence intercellular communication and have potential application as prognostic biomarkers for aneurysmal SAH patients (Sheng et al., 2023). Here we summarize the predictive significance of exosomes as biomarkers (Table 2). However, it is worth noting that most of the existing predictive studies can only demonstrate the detection of exosome-derived miRNAs as a result of SAH, rather than their causation. Therefore, further exploration is needed to determine its clinical application.

**TABLE 2:** Depict the exosomal miRNA’s diagnostic and prognostic value, with their application range.

Exosomal miRNA	Site	Tendency	Clinical significance
miR-603 (Li et al., 2022)	CSF	Improve	It has predictive significance in the context of vascular smooth muscle dysfunction and SAH.
miR-152-3p (Li et al., 2022)	CSF	Reduce	Distinguishes patients with UA from patients with hydrocephalus but also predicts SAH in patients with intracranial aneurysms.
miR-369-3p and miR-410-3p (Sheng et al., 2023)	Plasma	Reduce	It may affect intercellular communication and serve as a prognostic biomarker for aSAH
miR-193b-3p and miR-486-3p (Sheng et al., 2023)	Plasma	Improve	
miR-630 (Sun et al., 2019)	BCSF	Reduce	It indicates brain microvascular endothelial cells (BMECs) and brain microcirculation disturbance
miR-145 family, miR-29 family, etc (Liao et al., 2020)	Plasma	Improve	It may be a predictor of intracranial aneurysm and SAH
miR-27a-3p, miR-451a (Stylli et al., 2017)	CSF	Improve	It is important to identify potential vasospasm after SAH
miR-516a-5p, miR-566, miR-1197 (Stylli et al., 2017)	CSF	Reduce	May cause vasospasm

### 5.2 Therapeutic effects of MSC-derived exosomal miRNA on SAH

Mesenchymal stem cells are multipotent stem cells that possess self-renewal and multidirectional differentiation capabilities, and are present in various tissues including bone marrow, bone trabecule, skeletal muscle, and umbilical cord (Zhang et al., 2014). A plethora of studies indicate that MSCs can exert a neuroprotective effect through the delivery of exosomal miRNAs, and MSC-based therapy may constitute an important therapeutic approach for SAH in the future.

Exosomal miRNAs released from MSCs play a crucial role in SAH therapy through direct and indirect mechanisms. Firstly, exosomal miRNAs derived from MSCs exhibit anti-apoptotic effects. Brain-derived neurotrophic factor (BDNF) is a crucial factor that binds to the tropomyosin-related receptor kinase B receptor, facilitating nerve cell development and repair, regulating neural plasticity, and influencing emotional and cognitive functions (Colucci-D’Amato et al., 2020). Additionally, recent research has revealed the potential of BDNF in alleviating brain edema due to its ability to reduce BBB permeability, inhibit inflammatory responses, and enhance lymphangiogenesis and fluid absorption in brain tissue (Yan et al., 2022). Therefore, the introduction of exogenous miRNA-206 obtained from human umbilical cord-derived mesenchymal stem cells (hucMSCs) has exhibited promising outcomes by targeting BDNF, which leads to the alleviation of cerebral edema and the suppression of neuronal apoptosis (Zhao et al., 2019). Secondly, exosomal miRNAs derived from MSCs possess anti-inflammatory properties that counteract neuroinflammation induced by SAH. For example, exosomal miR-26b-5p from hucMSCs demonstrates the ability to reduce inflammation and alleviate neurological symptoms by modulating the p38 MAPK/STAT3 signaling pathway (Liu et al., 2021). Additionally, exosomal miR129-5p derived from bone marrow-derived MSCs exerts anti-inflammatory and anti-apoptotic effects, thus alleviating EBI in SAH (Xiong et al., 2020). Furthermore, MSC-derived exosomal miRNAs also contribute to the improvement of cerebral vasospasm. Among them, exosomal miR-126 released by MSCs promotes angiogenesis and inhibits vasospasm progression by targeting molecules involved in vascular smooth muscle contraction and endothelial cell function (Xi et al., 2017; Fu et al., 2019; Liu et al., 2022).

The utilization of exosomal miRNAs derived from MSCs holds great promise in the treatment of SAH. The ability of exosomes to deliver miRNAs to target cells opens new avenues for disease research and treatment, facilitating neuroprotection, alleviation of neuroinflammation, and prevention of cerebral vasospasm. Nonetheless, further investigations are needed to fully elucidate the underlying mechanisms and pathways involved in the therapeutic effects of MSC-derived exosomal miRNAs. Additionally, optimizing the isolation, purification, and delivery methods of exosomes holds significant potential for translating these research findings into effective clinical therapies for SAH.

### 5.3 Additional therapeutic value of exosomal miRNA

**FIGURE 2:**
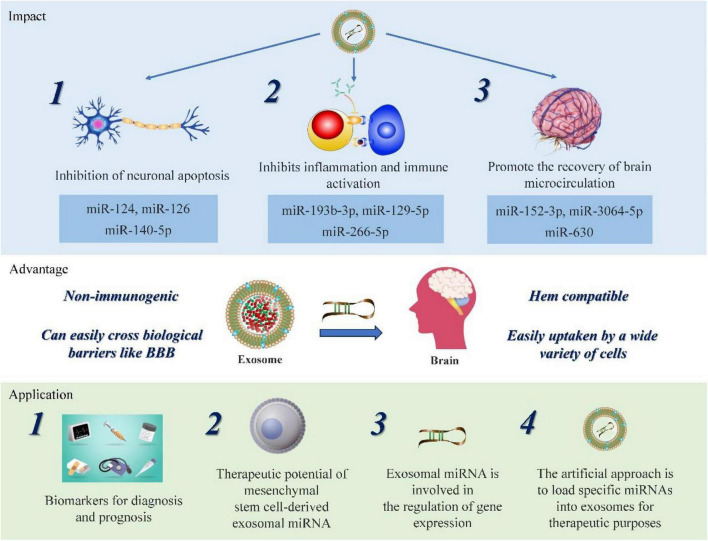
Highlight the potential therapeutic value and impact of exosomal miRNA in SAH. The figure highlights the potential clinical value and advantages of exosomal miRNAs following SAH. Upon the occurrence of SAH, exosomal miRNAs primarily participate in inhibiting neuronal apoptosis, suppressing inflammation and immune activation, and facilitating the restoration of brain microcirculation. Compared to other targeted molecules, exosomal miRNAs offer unique benefits, such as low immunogenicity, facile crossing of the BBB, and easy uptake by various tissues or cells. These characteristics confer a unique advantage for targeted therapies in SAH. Exosomal miRNAs have also been identified as diagnostic biomarkers for SAH and hold promise for gene regulation interventions and other clinical applications.

In addition to the discussed therapeutic value, exosomal miRNAs have various other potential uses, such as protecting the BBB and functioning as gene or drug transfer vectors (Figure 2).

Recent studies have emphasized that protecting the blood brain barrier (BBB) is a crucial avenue for improving the prognosis of SAH, and the development of suitable targeted drugs and therapies has become an urgent task. SIRT6, a histone deacetylase expressed in mature neurons essential for maintaining gene stability, has been found to preserve the integrity of the BBB and prevent ischemic stroke. Wang et al. (2023) investigated the potential mechanisms underlying the interaction between exosomal miR-3064-5p and SIRT6 and discovered that exosome miR-3064-5p may protect BBB integrity in SAH patients by inhibiting the SIRT6/PCSK9 pathway. This finding may provide a theoretical basis and potential therapeutic strategy for SAH.

Advancements in nanotechnology have tentatively resulted in the application of nanomaterials in hemorrhagic stroke therapy, including inorganic nanomaterials, polymer-based nanomaterials, lipid-based nanomaterials, and exosome gel systems, etc. (Dilnawaz et al., 2018; Su and Kang, 2020). Exosomes are a class of nanoscale structures with bilayer membranes whose interior can serve to load therapeutic molecules. Exosomes are characterized by low immunogenicity, strong protective ability, and the potential for effective BBB penetration (Quah and O’Neill, 2005; Batrakova and Kim, 2015; Zheng et al., 2019). Furthermore, due to their miRNA and neuroprotective protein content, they have shown considerable promise in anti-inflammatory, hemostatic and neuroprotective therapeutic paradigms.

## 6 Future perspectives

Exosomes possess various advantages, including high safety, low immunogenicity, capability to cross the BBB, and high efficiency of action (Katsuda et al., 2013). Exosomal miRNA holds significant potential in the diagnosis, treatment, and prognosis of SAH. This area merits further research. However, several obstacles and challenges limit the implementation of exosome miRNA-based therapy. First, although several proteins, RNAs, lipids, and metabolites have been identified in exosomes, limited information exists regarding their functions and sorting mechanisms. Despite the significance of exosomal miRNA as one of the most important substances in exosomes, our current understanding is restricted to its level of impact; further studies are necessary to comprehend its production mechanism and target action. Second, due to their role as intercellular communication agents, exosomes have strict requirements for the extracellular environment and their transport is complex. Thirdly, in bioengineering, urgent attention is required for challenges such as establishing relevant concentration control standards, identifying transportation routes accurately, and exploring production and storage methods. These issues involve the ability to generate, preserve, and transport exosomal miRNAs on a large scale, which plays a crucial role in the clinical application of exosomal miRNA. Soon, nanotechnology and genetic engineering could facilitate the development of exosomal miRNAs or their bioengineered counterparts, leading to promising clinical applications.

In summary, despite the challenges discussed earlier, the potential and significance of exosomal miRNA therapy for the treatment and management of SAH cannot be overlooked. Novel strategies and treatments currently being developed in this field hold immense promise and have the potential to significantly improve overall patient outcomes and survival rates. Further research into the sorting mechanism, target action, production, and storage of exosomal miRNA is essential and expected to make significant contributions in the future.

## Author contributions

LL: Writing – original draft. HW: Writing – original draft. DW: Data curation, Software, Writing – original draft. MY: Data curation, Validation, Writing – original draft. YG: Data curation, Supervision, Writing – original draft. MZ: Writing – review and editing. LW: Writing – review and editing.
